# Chondrome para-articulaire du genou

**DOI:** 10.11604/pamj.2015.20.320.6585

**Published:** 2015-04-02

**Authors:** Soufiane Guelzim, Ahmed Bardouni

**Affiliations:** 1 Service de Chirurgie Orthopédique et Traumatologie, CHU Ibn Sina, Rabat, Maroc

**Keywords:** Genou, chondrome, tumeur para-articulaire, knee, chondroma, Para-articular tumor

## Image en medicine

Les chondromes para-articulaires sont des tumeurs cartilagineuses bénignes qui se développent près des articulations.

L’étiologie de cette lésion n'est pas bien connue, mais elle résulte probablement de traumatismes répétés qui peuvent être à l'origine d'une métaplasie de cellules mésenchymateuses extra-synoviales.

Nous décrivons des images cliniques, paracliniques et per opératoires d'un chondrome para-articulaire du genou droit chez un jeune patient de 18 ans sans antécédents. Le patient avait consulté pour une tuméfaction douloureuse infra-patellaire du genou droit évoluant progressivement depuis 4 ans, sans notion de traumatisme. L'examen clinique a objectivé une masse ovoïde de 4cm de diamètre en regard du tendon rotulien, de consistance dure, mobile par rapport au plan superficiel et fixe par rapport au plan profond, il n'y avait pas de signes inflammatoires associés, l'examen général montre un patient longiligne avec une hyperlaxité constitutionnelle.

Les radiographies standard du genou droit face et profil ont montré une opacité au niveau des parties molles infra-patellaires, de grande taille mais bien limitée en regard du tendon rotulien, avec beaucoup de calcifications évoquant sa nature cartilagineuse. Par ailleurs, il n'y avait pas de lésions osseuses.

L'IRM a évoqué la nature cartilagineuse de la lésion et a précisé ses rapports intimes avec le tendon rotulien qui est refoulé en dehors. La tumeur est en hypo-signal en T1 et en hyper-signal en T2, le reste de l'examen est normal.

Par une voie d'abord para-patellaire médiale, l'exploration a mis en évidence une tumeur cartilagineuse bien limitée extra synoviale au sein du hoffa et adhérente au tendon rotulien. Nous avons réalisé la résection marginale de cette tumeur en respectant les fibres du tendon rotulien. Le diagnostic de chondrome para-articulaire a été confirmé par l'examen anatomo-pathologique.

Les suites post opératoires ont été simples.

Avec un recul de 2 ans, il n'y a pas de récidive de la tumeur et la mobilité du genou est normale.

**Figure 1:**
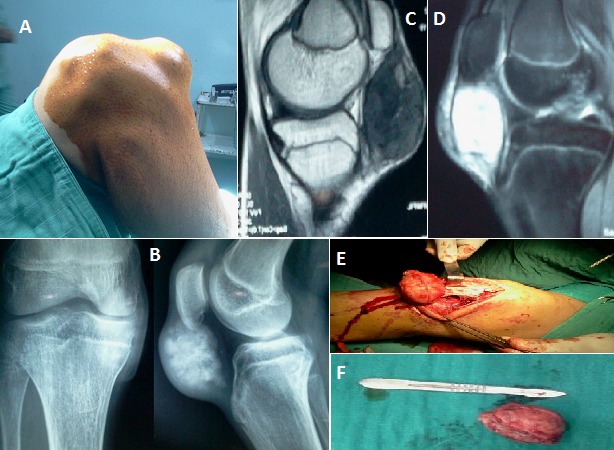
(A): image clinique: masse infra-patellaire du genou droit étendue de la pointe de la rotule à la TTA; (B): radiographie standard du genou droit face + profil: image infra-patellaire bien limitée avec calcifications évoquant la nature cartilagineuse de la tumeur; (C): IRM du genou droit: tumeur ovoïde en hypo-signal en T1; (D): IRM du genou droit: Tumeur ovoïde en hyper-signal en T2; (E): résection marginale de la tumeur facilement décollée sans lésions du tendon rotulien; (F): pièce opératoire: étude anatomopathologique

